# Canonical and Non-Canonical Activation of NLRP3 Inflammasome at the Crossroad between Immune Tolerance and Intestinal Inflammation

**DOI:** 10.3389/fimmu.2017.00036

**Published:** 2017-01-25

**Authors:** Carolina Pellegrini, Luca Antonioli, Gloria Lopez-Castejon, Corrado Blandizzi, Matteo Fornai

**Affiliations:** ^1^Department of Clinical and Experimental Medicine, University of Pisa, Pisa, Italy; ^2^Manchester Collaborative Centre for Inflammation Research, University of Manchester, Manchester, UK

**Keywords:** canonical, non-canonical, NLRP3, bowel inflammation, intestinal homeostasis, immune system, enteric microbiota

## Abstract

Several lines of evidence point out the relevance of nucleotide-binding oligomerization domain leucine rich repeat and pyrin domain-containing protein 3 (NLRP3) inflammasome as a pivotal player in regulating the integrity of intestinal homeostasis and shaping innate immune responses during bowel inflammation. Intensive research efforts are being made to achieve an integrated view about the protective/detrimental role of canonical and non-canonical NLRP3 inflammasome activation in the maintenance of intestinal microenvironment integrity. Evidence is also emerging that the pharmacological modulation of NLRP3 inflammasome could represent a promising molecular target for the therapeutic management of inflammatory immune-mediated gut diseases. The present review has been intended to provide a critical appraisal of the available knowledge about the role of canonical and non-canonical NLRP3 inflammasome activation in the dynamic interplay between microbiota, intestinal epithelium, and innate immune system, taken together as a whole integrated network regulating the maintenance/breakdown of intestinal homeostasis. Moreover, special attention has been paid to the pharmacological modulation of NLRP3 inflammasome, emphasizing the concept that this multiprotein complex could represent a suitable target for the management of inflammatory bowel diseases.

## Introduction

A growing body of evidence highlights the relevance of the nucleotide-binding oligomerization domain leucine rich repeat and pyrin domain-containing protein 3 (NLRP3) inflammasome in the pathophysiology of several autoinflammatory syndromes [i.e., cryopyrin-associated autoinflammatory syndromes (CAPS), Schnitzler’s syndrome], as well as metabolic and/or inflammatory disorders (i.e., obesity, atherosclerosis, type 2 diabetes, gout, and intestinal inflammation) (1–3). In the setting of intestinal microenvironment, NLRP3 inflammasome plays a pivotal role both in regulating the integrity of intestinal homeostasis and in shaping innate immune responses during bowel inflammation (3). In particular, the NLRP3 inflammasome, through the adaptor protein apoptosis-associated speck-like protein (ASC), recruits and activates caspase-1 leading to processing and release of IL-18 and IL-1β. These are two key cytokines involved both in the control of immune tolerance and support to immune and tissue events occurring in the presence of inflammation (4). This pathway is currently designated as “canonical NLRP3 inflammasome activation.” In addition, a “non-canonical NLRP3 inflammasome activation,” which depends on caspase-11 in mice (human orthologs are caspase 4 and caspase 5), has been described to be pivotal in the maintenance of intestinal immune homeostasis (5).

Several lines of preclinical evidence have unraveled a dual role of NLRP3 inflammasome in the pathogenesis of bowel inflammation (6). In particular, some studies showed a regulatory and reparative role of NLRP3 in the maintenance of immune tolerance and epithelial barrier integrity (7, 8). Conversely, others reported that the overactivation of NLRP3 inflammasome during intestinal inflammation is associated with a breakdown of intestinal immune balance, with consequent detrimental effects to the host (9). In this context, research efforts are currently being focused on a better understanding of the role of canonical and non-canonical NLRP3 inflammasome in the pathophysiology of intestinal inflammation.

Based on the above background, the present review has been intended to provide an integrated and critical appraisal of the available knowledge about the protective or detrimental role of canonical and non-canonical NLRP3 inflammasome activation in the maintenance of intestinal homeostasis as well as in sustaining the pathophysiological events underlying bowel inflammation. Special attention has been paid to point out how NLRP3 inflammasome influences the dynamic interplay between microbiota, intestinal epithelium, and innate immune system, as well as how the pharmacological modulation of this enzymatic complex could represent a suitable strategy in the management of inflammatory bowel diseases (IBDs).

## Mechanisms of Canonical and Non-Canonical NLRP3 Inflammasome Activation

**Figure 1:**
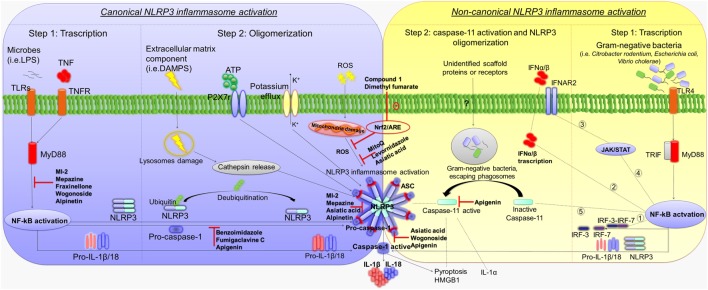
**Mechanisms of canonical and non-canonical NLRP3 inflammasome activation**. Diagram showing the canonical and non-canonical NLRP3 inflammasome activation, and representation of the molecular mechanisms through which several compounds inhibit NLRP3 activation and counteract intestinal inflammation. Left panel: first step of canonical NLRP3 inflammasome activation by TLRs–MyD88 and/or TNFR, which activate pro-IL-1β and NLRP3 transcription *via* NF-κB activation. The second step results in NLRP3 inflammasome oligomerization, leading to caspase-1 activation as well as IL-1β and IL-18 release. Extracellular ATP, degradation of extracellular matrix components, increase in potassium efflux, ROS, cathepsin activation, and deubiquitination promote NLRP3 inflammasome oligomerization and activation. Caspase-1 activation promotes also pyroptosis and HMGB1 release. Right panel: first step of non-canonical NLRP3 inflammasome activation. Gram-negative bacteria (i.e., *Citrobacter rodentium, Escherichia coli*, and *Vibrio cholerae*) activate the TLR4–MyD88 and TRIF pathways, with consequent nuclear translocation of NF-κB, which promotes the transcription of IL-1β, IL-18, and NLRP3 as well as IRF-3 and IRF7 genes. The IRF3–IRF7 complex (1) elicits the expression of IFN-α/β (2) that binds the IFNAR1/IFNAR2 receptor (3), leading to activation of the JAK/STAT pathway (4) and transcription of caspase-11 gene (5). In the second step, unidentified scaffold proteins or receptors induced by Gram-negative bacteria cleave and activate caspase-11, which induces pyroptosis as well as HMGB1 and IL-1α release, and promotes the activation of NLRP3-ASC-caspase-1 pathway. Abbreviations: NLRP3, nucleotide-binding oligomerization domain, leucine rich repeat and pyrin domain-containing protein 3; TLRs, toll-like receptors; MyD88, adaptor molecules myeloid differentiation primary response 88; TNFR, tumor necrosis factor receptor; NF-κB, nuclear factor-κB; ATP, adenosine triphosphate, ROS, reactive oxygen species; HMGB1, high mobility group box 1; TRIF, toll/IL-1 receptor homology (TIR)-domain-containing adapter-inducing interferon-β; IRF, interferon regulatory factor; IFN, interferon; IFNAR, interferon-α/β receptor; IL, interleukin; P2X, purinergic receptor 7; JAK/STAT, janus kinase/signal transducers and activators of transcription; NRF2/ARE, nuclear factor (erythroid-derived 2)-like 2/antioxidant response element.

Canonical NLRP3 inflammasome activation requires two parallel and independent steps: transcription and oligomerization (Figure 1) (10). The first step is regulated by innate immune signaling, mediated primarily by toll-like receptor (TLR)-adaptor molecules myeloid differentiation primary response 88 (MyD88) and/or cytokine receptors, such as the tumor necrosis factor receptor, which, in turn, activate pro-IL-1β and NLRP3 transcription *via* nuclear factor-κB (NF-κB) activation (11). The second step results in NLRP3 inflammasome oligomerization, leading to caspase-1 activation and, in turn, IL-1β and IL-18 processing and release (12). Various stimuli associated with infections, including an increase in extracellular adenosine triphosphate (ATP), extracellular osmolarity or pH alterations, β-amyloid fibers and degradation of extracellular matrix components, increase in potassium efflux, reactive oxygen species (ROS), cathepsin activation, and deubiquitination, can promote NLRP3 inflammasome oligomerization and activation by initiating assembly of a multiprotein complex consisting of NLRP3, the adaptor protein ASC, and pro-caspase-1. The recruitment of ASC is pivotal for the activation of pro-caspase-1 into its cleaved form (13–18). Caspase-1 activation promotes also, independently from IL-1β maturation, pyroptosis, a key defense mechanism against microbial infections, which blocks the replication of intracellular pathogens *via* cytoplasmic swelling and promotes phagocytosis of surviving bacteria (19–21). In particular, recent evidence has shown that caspase-1 cleaves the linker between the amino-terminal gasdermin-N and carboxy-terminal gasdermin-C domains in gasdermin D, an acid cytoplasmic protein, which plays a critical role in the process of pyroptosis (22, 23). Pyroptosis then promotes the release of additional cytosolic proteins, such as high mobility group box 1 (HMGB1) alarmin, a pro-inflammatory mediator significantly involved in the pathogenesis of several inflammatory chronic diseases (Figure 1) (24–26).

Besides canonical NLRP3 inflammasome activation, a non-canonical caspase-11-dependent NLRP3 activation has been characterized (Figure 1) (5). In particular, Gram-negative bacteria (i.e., *Citrobacter rodentium, Escherichia coli, Legionella pneumophila, Salmonella typhimurium*, and *Vibrio cholerae*) activate the TLR4–MyD88 and toll/IL-1 receptor homology-domain-containing adapter-inducing interferon-β (TRIF) pathways, with a consequent nuclear translocation of NF-κB, which in turn promotes the transcription of IL-1β, IL-18, and NLRP3 as well as interferon regulatory factor (IRF)-3 and IRF7 genes (27, 28). Subsequently, the IRF3–IRF7 complex elicits the expression of interferon (IFN)-α/β, which binds the IFN-α/β receptor 1 (IFNAR)/IFNAR2 receptor leading to activation of the JAK/STAT pathway and consequent transcription of caspase-11 gene (19, 29–31). In addition, binding of lipopolysaccharide (LPS) to caspase-11 and/or as-yet-unidentified scaffold proteins or receptors induced by Gram-negative bacteria, escaping phagosomes, have been shown to activate the effector functions of caspase-11 (32, 33). In particular, once activated, caspase-11 induces pyroptosis through cleavage of gasdermin, as well as HMGB1 and IL-1α release, and promotes IL-1β processing and release through activation of the NLRP3-ASC-caspase-1 pathway (Figure 1) (22, 23, 32).

These different NLRP3 activation processes occur independently. However, caspase-11 enhances the canonical caspase-1 processing and IL-1β/IL-18 production in the presence of specific stimuli (e.g., *cholerae toxin* or *E. coli*) (5, 22). In this setting, further *in vitro* experiments on cultured cells should be implemented to clarify the molecular mechanisms underlying the interplay between caspase-1 and -11 in promoting the canonical and/or non-canonical NLRP3 inflammasome activation.

## NLRP3 Inflammasome in the Pathophysiology of Bowel Inflammation

A dynamic interplay between enteric microbiota, intestinal epithelium, and mucosal immune system contributes to the maintenance of intestinal homeostasis (34). Indeed, dysbiosis, alterations of intestinal epithelial barrier and uncontrolled immune responses to pathogenic stimuli represent the main factors implicated in the pathogenesis of bowel inflammation. IBDs, including Crohn’s disease and ulcerative colitis, comprise chronic and relapsing inflammatory disorders that affect the gastrointestinal tract (35). In this context, NLRP3 inflammasome has been found to act as a key player both in the maintenance and breakdown of intestinal immune tolerance. Indeed, through the regulation of intestinal epithelial and immune innate cells (monocytes, macrophages and dendritic cells), it contributes to maintaining intestinal homeostasis, while sustaining also the pathophysiological events underlying bowel inflammation (36). However, despite more than a decade has passed since the discovery of inflammasomes, the role of NLRP3 inflammasome in the intestinal homeostasis as well as in the pathophysiology of bowel inflammation remains multifaceted and controversial (6). A number of preclinical investigations have attempted to unravel the role played by NLRP3 inflammasome in this setting. Accordingly, current data on the involvement of canonical and non-canonical NLRP3 pathways in the pathophysiology of bowel inflammation are addressed in the following section.

### Canonical NLRP3 Inflammasome Activation

**Figure 2:**
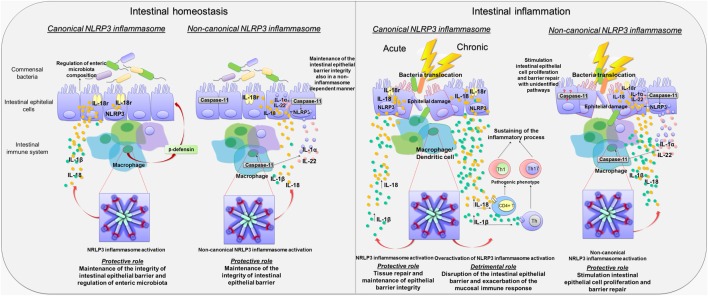
**Canonical and non-canonical activation of nucleotide-binding oligomerization domain leucine rich repeat and pyrin domain-containing protein 3 (NLRP3) inflammasome pathways in intestinal homeostasis and inflammation**. Diagram showing the role of canonical and non-canonical activation of NLRP3 inflammasome pathways in intestinal homeostasis and inflammation. Left panel: Canonical and non-canonical NLRP3 inflammasome activation in intestinal homeostasis. Canonical NLRP3 inflammasome activation plays a key role in the maintenance of the integrity of intestinal epithelial barrier as well as in the enteric microbiota composition through the release of IL-18 by macrophages and intestinal epithelial cells, the regulation of crypt bactericidal capacity, and the release of colonic β-defensin by macrophages. Likewise, non-canonical NLRP3 inflammasome activation contributes to maintain the integrity of intestinal epithelial barrier through IL-18 release by macrophages and intestinal epithelial cells. In addition, caspase-11 contributes, in a NLRP3-independent manner, to the maintenance of intestinal homeostasis promoting the release of Il-1α and IL-22. Right panel: Canonical and non-canonical NLRP3 inflammasome activation in intestinal inflammation. In the acute phase of inflammation, canonical NLRP3 inflammasome activation promotes the release of IL-1β and IL-18, contributing to tissue repair and maintenance of epithelial barrier integrity. Conversely, in the chronic phase of inflammation, canonical NLRP3 inflammasome overactivation is associated with an increase in IL-1β and IL-18 release that is harmful to the host. In addition, IL-1β and IL-18 release induce the differentiation of T cells into pathogenic Th1 and Th17 phenotypes, which contribute to sustain the inflammatory response. Non-canonical NLRP3 inflammasome activation plays a protective role during bowel inflammation likely *via* IL-18 release that stimulates intestinal epithelial cell proliferation and barrier repair. In addition, the release of IL-22 and IL-1α contributes to the repair of intestinal epithelial barrier. However, caspase-11 contributes also to promote intestinal epithelial cell proliferation and barrier repair by recruitment of yet unidentified inflammasome-independent pathways.

In an attempt of understanding the role of canonical NLRP3 inflammasome in the pathophysiology of bowel inflammation, several efforts have been made to implement research on the effects of NLRP3 gene deletion and its components on immune and non-immune cell activity, as well as on pathophysiological events downstream its activation in preclinical models of colitis (see Table 1). Two initial reports showed that NLRP3 plays a key role in the regulation of intestinal homeostasis, maintaining the epithelial barrier integrity and reducing mortality during experimental colitis (7, 37). In particular, *Nlrp3*^−/−^, *Asc*^−/−^, and *caspase-1*^−/−^ mice were found to be more susceptible to colitis induced by dextran sodium sulfate (DSS) and 2,4,6-trinitrobenzenesulfonic acid (TNBS), both characterized by body weight loss, diarrhea, rectal bleeding, and mortality, suggesting a protective role of NLRP3 inflammasome in the digestive tract. Such a favorable action was ascribed to the ability of NLRP3 of inducing IL-18 release, a crucial mediator in the repair of colonic mucosal barrier that, through binding IL-18 receptors on intestinal epithelial cells, exerts a restorative effect on the enteric epithelium (Figure 2). In addition, *Nlrp3*^−/−^ mice showed an elevation of nitric oxide (NO) levels, likely resulting from an increase in inducible NO synthase (iNOS) activity, and a decrease in the anti-inflammatory IL-10 cytokine and protective growth factor TGF-β expression, thus suggesting the ability of NLRP3 to regulate the production of pro- and anti-inflammatory mediators in the presence of bowel inflammation (7). However, the molecular mechanisms underlying NLRP3 inflammasome-dependent regulation of these inflammatory factors remain to be determined. In the same paper, the authors showed also that NLRP3 activation modulated the activity and trafficking of neutrophils as well as leukocyte recruitment. In particular, NLRP3-deficient neutrophils showed a pattern of altered migration, attenuated chemotactic responses, and enhanced spontaneous apoptosis (7). These findings support the view that NLRP3 inflammasome acts in a reparative key, regulating neutrophil and leukocyte phagocytic activity.

**Table 1:** **Summary of current pre-clinical evidence supporting the differential role of NLRP3 inflammasome in intestinal inflammation**.

Animal model	Genetic phenotype	Treatment/timing	Outcome	Role of NLRP3	Reference
DSS	*Nlrp3*^−/−^, *Asc*^−/−^, and *caspase-1*^−/−^	2.5% (w/v)/6 days	✓ Body weight loss ✓ Diarrhea ✓ Rectal bleeding mortality	Protective	(7)
TNBS	*Nlrp3*^−/−^, *Asc*^−/−^, and *caspase-1*^−/−^	(30 mg/mL)/3 days	✓ Body weight loss ✓ Diarrhea ✓ Rectal bleeding mortality	Protective	(7)
DSS	*Nlrp3*^−/−^, *Asc*^−/−^, and *caspase-1*^−/−^	✓ 3% (w/v)/5 days and sacrifice at day 7 ✓ 3% (w/v)/7 days and sacrifice at day 9	✓ Disruption of the intestinal epithelial barrier ✓ Increase in mucosal permeability ✓ Bacterial translocation ✓ Systemic dissemination	Protective	(37)
DSS	*Nlrp3*^−/−^, *Asc*^−/−^, *caspase-1*^−/−^	2% (w/v)/9 days	✓ Less severity of colitis ✓ Reduced pro-inflammatory cytokines levels	Detrimental	(9)
DSS	*Nlrp3*^−/−^, *Asc*^−/−^, *caspase-1*^−/−^	2% (w/v)/9 days	✓ Less severity of colitis ✓ Reduced pro-inflammatory cytokines levels	Detrimental	(41)
*IL-10*^−/−^	*IL-10*^−/−^	n.a.	✓ Increase in colonic IL-1β and IL-17 levels	Detrimental	(42)

*DSS, dextran sodium sulfate; TNBS, 2,4,6-trinitrobenzenesulfonic acid; w/v, weight/volume; IL-1β, interleukin-1beta; IL-17, interleukin-17*.

Consistent with the above results, Zaki et al. observed that the induction of colitis in *Nlrp3*^−/−^ mice was associated with a disruption of intestinal epithelial barrier and an increase in mucosal permeability as compared to DSS wild-type (WT) mice, with consequent bacterial translocation into the mucosa and systemic dissemination (37). Such detrimental effect on the intestinal epithelial barrier, besides a decrease in the inflammasome-dependent IL-18 cytokine release, resulted from the ability of NLRP3 to regulate crypt bactericidal capacity and the expression of colonic β-defensin, an antimicrobial peptide released by macrophages implicated in the resistance of epithelial surfaces to microbial colonization (Figure 2) (7). These findings highlight the relevance of NLRP3 in modulating the interplay between intestinal epithelium and innate immune cells, suggesting a key role of NLRP3 inflammasome in the maintenance of the integrity of intestinal epithelial barrier as well as in orchestrating the mucosal innate immune response during inflammation. However, further investigations should be implemented to identify the exact mechanism through which NLRP3 modulates the intestinal epithelium–innate immune system interplay both under physiological conditions and in the presence of bowel inflammation.

Besides the regulation of non-immune and immune cells activity, NLRP3 inflammasome influences also the composition of enteric microbiota (7, 38). In particular, fecal microbiota in NLRP3^−/−^ mice did dramatically differ, in terms of load and species, from WT mice, and such microbial shifts occurred in *Nlrp3*^−/−^ mice prior to the induction of colitis, suggesting that a reduced inflammasome functionality is associated with enteric bacterial dysbiosis (37). Taken together, these findings expand further available knowledge about the regulatory role of inflammasome in homeostasis, since it appears to coordinate a dynamic interplay between gut microbiota and epithelium–innate immune system, contributing to the maintenance of intestinal microenvironment integrity (Figure 2).

Several lines of evidence point out the concept that deficiencies in NLRP3 inflammasome components can protect mice from DSS-induced colitis (9, 39). In particular, in a study by Bauer et al., DSS *Nlrp3*^−/−^ mice developed a less severe colitis and produced lower levels of pro-inflammatory cytokines as compared with DSS WT mice. In addition, the pharmacological inhibition of caspase-1 with pralnacasan protected from colonic mucosal damage as with NLRP3 deficiency, suggesting that NLRP3 inflammasome contributes to the pathophysiology of intestinal inflammation and that NLRP3 blockade could represent a viable pharmacological strategy for the management of bowel inflammation (39, 40). Different findings about the different roles of NLRP3 inflammasome in bowel inflammation might be ascribed to different experimental conditions. For instance, in reports showing a protective and regulatory action of NLRP3, experiments were performed at the seventh day after 5 days of 3% DSS treatment and 2 days without DSS exposure, or 7 days of continued 2.5% DSS exposure. By contrast, in the paper by Bauer et al., describing a detrimental role of NLRP3 in colitis, mice received 2% DSS for 9 days. It is therefore conceivable that, extending DSS exposure, the overactivation of NLRP3 becomes detrimental for the intestinal microenvironment. Indeed, 6 days after colitis induction, the histopathological score was significantly reduced in DSS NLRP3^−/−^ mice, as compared with DSS WT (39).

Interestingly, in a subsequent paper, Bauer et al. hypothesized a putative role of NLRP3 inflammasome both in the innate and adaptive immune response. In particular, unlike intestinal epithelial cells, where inflammasome-induced IL-18 release promoted a mucosal repair after DSS-induced damage, NLRP3-induced IL-18 release from lamina propria macrophages and dendritic cells elicited the activation and differentiation of CD4^+^ T cells into the Th1 pro-inflammatory phenotype (Figure 2) (41). The relevance of NLRP3 in the modulation of immune cell differentiation was further confirmed by observing that lamina propria dendritic cells in NLRP3^−/−^ mice expressed a tolerogenic phenotype (CD103^+^ DC) both under physiological and inflammatory conditions, which may, at least in part, explain the reduced susceptibility of *Nlrp3*^−/−^ mice to colitis (41). However, the molecular mechanisms through which NLRP3 triggers the differentiation of dendritic cells into a pro-inflammatory phenotype are still unclear and deserve further investigations.

In support of the above results, showing a detrimental role of NLRP3 in bowel inflammation, Zhang et al. (9) observed a causative link between NLRP3 inflammasome activation and development of chronic intestinal inflammation, showing that the increase in colonic IL-1β levels in *IL-10*^−/−^ mice promoted IL-17 release, known to contribute to the pathogenesis of chronic colitis both in animal models and IBD patients (42). Treatment with IL-1 receptor antagonist or caspase-1 inhibitors suppressed IL-1β and IL-17 production, thus ameliorating spontaneous colitis in *IL-10*^−/−^ mice. In this setting, it appears that the lack of anti-inflammatory IL-10 cytokine triggers unknown molecular mechanisms that could influence IL-1β release through gene transcription and/or direct regulation of canonical caspase-1-dependent inflammasome activation (9). Based on these findings, the authors hypothesized that, in the absence of anti-inflammatory IL-10 cytokine, intestinal inflammasomes undergo a condition of prolonged activation, leading to an uncontrolled and aberrant inflammasome-mediated immune response that contributes to the development of chronic colitis (9). Therefore, since IL-10 appears to modulate inflammasome activation, an *in vivo* pharmacological modulation of IL-10 in animal models of hapten-induced colitis could help to unravel the mechanisms underlying the negative regulation of inflammasome by IL-10.

Another considerable issue pertains to the relationship between NLRP3 and gut microbiota. Indeed, Bauer et al. (41) observed that cohousing of *Nlrp3*^−/−^ mice with WT animals abrogated the protective effect of NLRP3 deficiency during colitis, and increased DSS susceptibility. Based on these results, it is conceivable that changes in enteric bacterial composition and a condition of NLRP3 hypo-functionality could contribute to the pathophysiology of bowel inflammation (41). Therefore, although these findings are in contrast with previous observations, showing a protective role of NLRP3 during colitis (37), both highlight the relevance of NLRP3-enteric microbiota interplay in the maintenance of intestinal homeostasis.

### Non-Canonical NLRP3 Inflammasome Activation

Besides canonical NLRP3 inflammasome activation, over the last years, a pivotal role in the pathophysiology of intestinal inflammation has been proposed also for non-canonical caspase-11-dependent NLRP3 inflammasome activation. In particular, caspase-11, widely expressed in both hematopoietic- and non-hematopoietic cells, including macrophages and epithelial cells, once activated by Gram-negative bacteria, promotes NLRP3 inflammasome assembly and subsequent release of IL-1β, IL-18 and regulates IL-1α and HMGB1 release and pyroptosis (43, 44).

The contribution of caspase-11 to NLRP3 inflammasome activation has been initially investigated in animal models of acute exposure to enteric bacteria, sepsis, and endotoxic shock (45, 46). Recently, several lines of evidence have shown that caspase-11-induced inflammasome activation plays a protective role during intestinal inflammation (8, 47). In particular, Williams et al. (8) observed that *Casp11*^−/−^ mice displayed a significantly increased morbidity, colonic tissue damage, and leukocyte infiltration following DSS exposure, thus suggesting an increased susceptibility to DSS-induced colitis that was ascribed to a decrease in colonic inflammasome-induced IL-1β and IL-18 release (8). DSS *Casp11^−/−^* mice showed also a significant increase in caspase-1 expression, which, however, was not associated with an increased inflammasome activity (8). In support of the protective role of caspase-11, it has been observed that both chimeric *Casp11^−/−^* mice, receiving WT bone marrow (WT → *Casp11^−/−^*), and chimeric WT mice, receiving *Casp11^−/−^* bone marrow (*Casp11^−/−^* → WT), were more sensitive to DSS-induced colitis as compared to WT mice receiving WT bone marrow. In particular, chimeric WT → *Casp11^−/−^* or *Casp11^−/−^* → WT DSS mice displayed a significantly increased histopathological damage, epithelial tethering, large areas of erosion, extensive areas of ulceration, enhanced inflammatory cell infiltration, and increased crypt atrophy as compared with chimeric DSS WT → WT animals (8). A proposed mechanism, underlying the protective role of caspase-11-induced inflammasome activation, calls into play its ability of regulating the release of IL-18, IL-22, and IL-1α cytokines, known to promote intestinal epithelial cell proliferation and barrier repair (47). Indeed, *Casp11^−/−^* DSS mice showed reduced colonic IL-18, IL-22, and IL-1α levels in comparison with WT DSS animals, thus suggesting that decreased levels of these cytokines, in particular IL-18, could contribute to the increase in epithelial barrier permeability, with consequent bacterial translocation into the lamina propria and exacerbation of the inflammatory response (48–50). Taken together, these findings suggest that caspase-11, *via* non-canonical inflammasome activation, regulates mucosal and epithelial barrier integrity during intestinal inflammation by increasing epithelial cell proliferation and inhibiting cell death (Figure 2).

The protective role of caspase-11-induced non-canonical NLRP3 inflammasome activation has been shown in a model of Gram-negative *C. rodentium* infection-induced colitis (51). In particular, in autophagy defective mice, made knock out for nucleotide-binding oligomerization domain-like receptors NLRs (NOD2) and recruit receptor interacting protein 2, infected with *C. rodentium*, the increase in oxidative stress activated the c-Jun N-terminal kinase (JNK) signaling that, in turn, increased caspase-11 expression and non-canonical NLRP3 inflammasome activation, with consequent protection of colonic epithelial barrier (51). These results corroborate previous findings, supporting the regulatory role of non-canonical NLRP3 inflammasome activation in the maintenance of intestinal homeostasis, and, most importantly, they show that, besides the TLR4–TRIF–IFN-β pathway, JNK signaling promotes non-canonical inflammasome activation. In this context, despite the observation that JNK signaling can be activated also by TLR stimulation (52), it is not clear whether the stimulation of TLR–MyD88–JNK pathway promotes caspase-11-dependent non-canonical NLRP3 inflammasome activation during intestinal inflammation. Therefore, there is still need to evaluate the role of this pathway in animal models of colitis.

At odds with the above data, Demon et al. (53) suggested that sensitivity to DSS colitis in *Casp11*^−/−^ mice is independent from caspase-1-induced canonical inflammasome activation, since the colonic levels of IL-1β and IL-18, as well as circulating HMGB1 in DSS *Casp11*^−/−^ mice, did not differ from DSS WT mice, suggesting that caspase-11 protects against colitis independently from inflammasome activation and, therefore, hypothesizing an unidentified pathway for caspase-11 in bowel inflammation (Figure 2) (53). These conflicting findings about the relevance of caspase-11-induced non-canonical inflammasome activation could result from different experimental designs, as well as from environmental variability. For instance, since caspase-11 is activated by Gram-negative bacteria, changes in gut microbiota composition could (i) influence hypo- or hyper-activation of caspase-11-induced non-canonical NLRP3 inflammasome, (ii) alter basal IFN production (54, 55), with subsequent changes in caspase-11 expression and/or function, or (iii) trigger yet unidentified molecular NLRP3-independent pathways involved in the pathophysiology of gut inflammation.

### Discussion

Current data allow to hypothesize that NLRP3 inflammasome can play both protective and detrimental roles in bowel inflammation, depending on the choice of colitis models and variations of commensal enteric microflora. In particular, in the model of DSS-induced acute colitis, which causes a direct damage to the epithelial barrier, with consequent stimulation of innate immune cells by commensal bacteria, infiltration of myeloid cells and massive inflammation, IL-1β and IL-18 appear to be essential for tissue repair and the maintenance of epithelial barrier integrity (56), thus suggesting a protective role of NLRP3. By contrast, in animal models of chronic colitis, the inflammasome-induced IL-1β release induces differentiation of T cells into pathogenic Th17 phenotypes, thus contributing to sustain the inflammatory process (9). Despite these conflicting and heterogeneous findings, it appears that, in the first acute phase of inflammation, the NLRP3 inflammasome acts as a key player to restore intestinal homeostasis. Conversely, in chronic colitis, where a disruption of the intestinal epithelial barrier and an exacerbation of the mucosal immune response occur, the overactivation of NLRP3 inflammasome results to be harmful to the host (Figure 2). However, the results from knockout mouse models, where there is a complete removal of NLRP3 protein complex, cannot be easily and fully translated into the clinical setting, since NLRP3 gene deletion might trigger unknown compensatory immune mechanisms that influence the disease outcome. Furthermore, it is also noteworthy that in animals with complete deletion of NLRP3 gene no distinction between canonical and/or non-canonical NLRP3 inflammasome activation can be made. Therefore, the *in vivo* pharmacological modulation of canonical NLRP3 inflammasome in more predictive animal models of colitis should be investigated, in order to clarify the role of this enzymatic complex in the pathophysiology of bowel inflammation. Data on the effects stemming from the pharmacological modulation of NLRP3 inflammasome are discussed in the following sections.

With regard to non-canonical NLRP3 inflammasome activation, the majority of current data suggest that caspase-11-dependent NLRP3 activation, although dispensable for caspase-1-inflammasome assembly, contributes to protection against DSS-induced colitis regulating the epithelial barrier integrity. However, owing to scarce, conflicting and heterogeneous findings, it remains unclear whether caspase-11, expressed in the colonic mucosa, plays a protective role also in intestinal inflammation independently from canonical NLRP3 inflammasome activation. Accordingly, there is a strong need for further experiments, aimed at evaluating how the simultaneous caspase-1 and caspase-11 gene deletion, as well as the pharmacological modulation of caspase-11, could interfere with NLRP3 assembly and consequently with the pathophysiology of bowel inflammation. In addition, given the relevance of intestinal microbiota in caspase-11 activation, extensive investigations are needed to evaluate whether changes in enteric bacteria composition could influence caspase-11 activity and consequent non-canonical inflammasome assembly.

## Pharmacological Modulation of NLRP3 Inflammasome in Bowel Inflammation

The involvement of inflammasome pathways in the pathophysiology of intestinal inflammation is fostering research on the potential therapeutic benefits, in terms of anti-inflammatory activity, resulting from the pharmacological targeting of NLRP3 inflammasome. At present, the majority of available studies have investigated the role of NLRP3 in several experimental models of colitis, displaying remarkable beneficial effects by the pharmacological modulation of this enzymatic complex (57–59). In particular, DSS-induced colitis has been largely employed, since in this model lysosomal damage and increased ROS levels can lead to an overactivation of NLRP3 inflammasome (39). Following DSS administration, surface molecules produced by microorganisms or other inflammatory factors (i.e., LPS) can promote also the first step of NLRP3 assembly, through the activation of NF-κB transcription, with subsequent increase in NLRP3 as well as pro-IL-1β and pro-IL-18 protein levels. Furthermore, DSS treatment is associated with an increase in extracellular ATP or bacterial toxins, which are able to stimulate caspase-1 activation directly, thereby releasing IL-1β and IL-18 (39, 58). Accordingly, several targets have been identified for inhibiting the assembly of NLRP3 (Figure 1).

A pioneering study by Dashdorj et al. (59) showed that MitoQ, a mitochondria-targeted derivative of the antioxidant ubiquinone, endowed with antioxidant and anti-apoptotic properties, exerted beneficial effects on experimental colitis through a decrease in colonic NLRP3 and caspase-1 expression, with consequent decrease in IL-1β and IL-18 release (59). The molecular mechanism underlying NLRP3 inflammasome blockade was proposed to depend on the ability of MitoQ to suppress ROS-induced dissociation of thioredoxin-interacting protein (TXNIP), from thioredoxin, thus inhibiting the interaction of TXNIP with NLRP3 (59). Indeed, although Masters et al. (60) showed that TXNIP is not essential to NLRP3 activation in bone marrow-derived macrophages primed with LPS and then stimulated with *S. aureus*, silica, or ATP, in the setting of colitis, where an increase in oxidative stress and activation of different inflammatory pathways occur, the dissociation of TXNIP could represent one of the mechanisms underlying NLRP3 activation (59).

The inhibition of mitochondrial ROS generation, as a suitable pharmacological target for inhibiting NLRP3 inflammasome assembly, has been confirmed by a subsequent study, showing that *in vivo* administration of levornidazole, the levo isomer of ornidazole generally used for protozoan infections, to DSS mice exerted enteric anti-inflammatory effects through the blockade of NLRP3 inflammasome assembly by suppression of ROS generation. These findings suggest that the blockade of NLRP3 upstream signaling could represent a suitable pharmacological target for the management of intestinal inflammation (61).

Consistent with the above data, two recent papers by Wang et al. (58) and Liu et al. (62) have reported that the inhibition of ROS formation exerted beneficial effects in colitis through the blockade of NLRP3 assembly (58, 62). In particular, these authors observed that two small molecules, 3-(2-oxo-2-phenylethylidene)-2,3,6,7-tetrahydro-1H-pyrazino-[2,1-a]isoquinolin-4(11bH)-one (compound 1) and dimethyl fumarate, promote the transcription of genes coding for various detoxification and antioxidant enzymes, through the activation of NFE-related factor 2 (Nrf2). The subsequent inhibition of ROS formation has been shown to exert inhibitory effects on NLRP3 assembly (58, 62). Indeed, after exposure to environmental or intracellular stresses, such as ROS, Nrf2 translocates into the nucleus and binds to antioxidant response elements (AREs), which in turn induce the production of cytoprotective enzymes, such as heme oxygenase 1, NAD(P)H quinine oxidoreductases, and glutathione *S*-transferases, that are pivotal to maintain optimal cellular functions (63). In this respect, the stimulation of Nrf2/ARE pathway could represent an indirect molecular target to inhibit NLRP3 inflammasome activation. However, further extensive investigations are needed to characterize the actual molecular mechanisms underlying the Nrf2–ROS–NLRP3 interplay and, most importantly, the correlation between Nrf2 stimulation and NLRP3 inhibition in the setting of bowel inflammation.

Besides targeting the inflammasome upstream signaling, NLRP3 blockade *via* caspase-1 inhibition has been shown also to exert anti-inflammatory effects in DSS mice (57, 64). In particular, a synthetic benzimidazole derivative and fumigaclavine C, a fungal metabolite, through the inhibition of caspase-1 activation, exerted beneficial effects on colonic inflammation reducing protein and mRNA levels of colonic TNF, IL-1β, and IL-17 pro-inflammatory cytokines (57, 64). However, even if both compounds have caspase-1 inhibition as ultimate goal, they influence intracellular signaling in different ways. Indeed, the benzimidazole derivative has been shown to inhibit MAPK and STAT1 signaling without interaction with NF-κB-mediated transcription, while fumigaclavine C was found to significantly interfere with NF-κB activation, STAT3 and STAT1 signaling. These findings suggest that the inhibition of the primary TRL–MyD88–NF-κB step involved in NLRP3 inflammasome activation, though at different steps of the intracellular cascade, represents a suitable pharmacological target for inhibiting the NLRP3 assembly, and therefore a promising strategy for treatment of bowel inflammation.

In further support of the above data, a recent paper by Liu et al. (65) showed that the inhibition of mucosa-associated-lymphoid-tissue lymphoma-translocation gene 1 (MALT1), a scaffold protein, which recruits the IκB kinase complex leading to release and activation of NF-κB, ameliorated clinical symptoms and histopathologic features of DSS-induced colitis through NF-κB and NLRP3 inhibition, thus interfering with both inflammasome activation steps (65, 66). In particular, treatment with two specific MALT1 inhibitors, MI-2 and mepazine, dose-dependently attenuated the symptoms of colitis in mice through a decrease in protein and mRNA levels of colonic TNF, IL-1β, IL-6, IL-18, IL-17A, and IFN-γ pro-inflammatory cytokines (66). The mechanisms underlying the inhibitory effects of MALT1 in DSS-induced colitis have been ascribed to the inhibition on NF-κB and NLRP3 inflammasome activation in macrophages, thus implying that MALT1-NF-κB signaling regulates NLRP3 inflammasome activation. However, since the NF-κB pathway is involved in the transcription of both pro- and anti-inflammatory mediators (i.e., IL-10 and TGF-β), extensive investigations are required to identify more selective targets to inhibit NLRP3 inflammasome oligomerization, thereby counteracting the pathophysiological events underlying NLRP3 activation, and attenuating bowel inflammation.

Of interest, several lines of evidence have shown that various NLRP3-targeting natural compounds are able to exert anti-inflammatory effects on DSS-induced colitis in mice. Guo et al. (66) observed that oral administration of asiatic acid, a natural triterpenoid compound, dose-dependently attenuated body weight loss, shortening of colon length, histopathologic scores, myeloperoxidase activity, and colonic TNF, IL-1β, IL-6, and IFN-γ levels in mice with DSS-induced colitis through the inhibition of NLRP3 inflammasome activation (67). In particular, the authors found that asiatic acid inhibited the upstream signaling of inflammasome oligomerization by suppressing mitochondrial ROS generation, as well as caspase-1 activation and inflammasome assembly. Likewise, treatment with fraxinellone, a natural lactone endowed with immunosuppressive activity, significantly reduced weight loss, diarrhea and colonic macroscopic damage, as well as myeloperoxidase, alkaline phosphatase, and colonic TNF, IL-1β, IL-6, and IL-18 levels in DSS-induced colitis mice (68). Such anti-inflammatory effects were ascribed to the inhibition of CD11b^+^ macrophage infiltration, as well as the decrease in mRNA levels for colonic macrophage-related proteins, including intercellular adhesion molecule 1 (ICAM1), vascular cell adhesion molecule 1 (VCAM1), iNOS, and cyclooxygenase-2 (COX-2), through NF-κB signaling and NLRP3 inhibition. These findings represent a point of novelty, since they support the view that the blockade of NLRP3 assembly can influence also the activation of infiltrating macrophages by inhibiting the release of intercellular adhesion molecules and pro-inflammatory mediators contributing to the inflammatory process.

Recent evidence has shown that several flavonoid derivatives exerted anti-inflammatory effects on colitis *via* NF-κB/NLRP3 inhibition (69–71). In particular, treatment with wogonoside, a glucuronide metabolite of the bioactive flavonoid wogonin, reduced significantly colonic NF-κB and NLRP3 expression, as well as caspase-1 expression and activity in mice with colitis, exerting beneficial effects on colonic inflammation (69). Likewise, the administration of alpinetin, a novel plant flavonoid isolated from *Alpinia katsumadai Hayata*, significantly attenuated diarrhea, colonic shortening, histological damage, and myeloperoxidase activity as well as colonic TNF and IL-1β expression in mice with DSS-induced colitis, likely by suppressing TRL4-NF-κB and NLRP3-ASC-caspase-1 signaling (70). However, the authors documented the ability of alpinetin of inhibiting NLRP3 activation in *in vitro* THP-1 cells, omitting the evaluation of alpinetin effects on NLRP3 activation in DSS mice.

The protective effects of flavonoids *via* NLRP3 inhibition have been shown also in a mouse model of DSS-induced colitis (71). In particular, a dietary apigenin (API) enrichment decreased the macroscopic and microscopic signs of colitis and reduced colonic PGE, COX-2, and iNOS expressions as well as serum matrix metalloproteinase (MMP-3) levels. In addition, API diet reduced IL-1β and TNF pro-inflammatory cytokine release in primary LPS-stimulated splenocytes. The beneficial effects of API on colonic inflammation result from the inhibition of both canonical and non-canonical NLRP3 inflammasome pathways, through the regulation of caspase-1 and caspase-11 enzyme expression and activity (71). Indeed, although caspase-11 has been proposed to mediate a protective role in the host during the acute phases of colitis, it appears to be detrimental in chronic inflammation, where it is significantly upregulated and promotes IL-1β and IL-18 release (8, 47). These findings demonstrate, for the first time, that the pharmacological blockade of both canonical and non-canonical NLRP3 activation could represent a suitable and promising pharmacological target for treatment of bowel inflammation. Nevertheless, the molecular mechanisms through which API can block canonical and non-canonical NLRP3 assembly remain to be clarified.

## Overall Conclusion and Future Directions

Studies aimed at characterizing the molecular mechanisms and downstream signaling underlying the canonical and non-canonical NLRP3 inflammasome activation have unraveled the pivotal and dual role of this enzymatic complex in the intestinal homeostasis. According to current information, NLRP3 regulates the integrity of intestinal mucosal barrier under physiological conditions, but it can shape also the immune response against commensal microbiota during bowel inflammation.

One considerable deficiency in our knowledge concerns how canonical and non-canonical NLRP3 inflammasome coordinate differently the dynamic interplays among gut microbiota–epithelium–innate immune system. For instance, it remains unclear whether alterations of enteric bacteria composition promote an abnormal caspase-1- and/or caspase-11-dependent NLRP3 inflammasome activation, or whether, *vice versa*, the overactivation of NLRP3 alters epithelial barrier integrity and gut microbiota with consequent alteration of intestinal homeostasis. In this context, given the high complexity of the NLRP3 inflammasome system, strong efforts are needed to better understand how canonical and non-canonical NLRP3 manage their downstream signaling to maintain or break down intestinal homeostasis. Nevertheless, despite current heterogeneous and conflicting evidence, beneficial effects resulting from the pharmacological modulation of NLRP3 in animal models of colitis have led to postulate that the overactivation of NLRP3 during bowel inflammation is detrimental for the host. Therefore, the blockade of NLPR3 activation could represent a suitable pharmacological approach for the management of inflammatory intestinal disorders.

A recent study by Coll et al. (72) showed that the pharmacological blockade of canonical and non-canonical NLRP3 activation with MCC950, a recognized selective, small molecule inhibitor of NLRP3, reduced IL-1β tissue levels and attenuated the severity of experimental autoimmune encephalomyelitis, an animal model of multiple sclerosis (72). Furthermore, MCC950 rescued neonatal lethality in a mouse model of CAPS, and it was effective in reducing IL-1β levels in human peripheral blood mononuclear cells from patients with Muckle–Wells syndrome, thus suggesting its putative therapeutic activity in NLRP3-associated syndromes, including autoinflammatory and autoimmune diseases (72). Based on these observations, testing MCC950 in animals with colitis (i.e., DSS, TNBS, or *IL-10*^−/−^) would allow a better characterization of the anti-inflammatory effects resulting from selective inhibition of canonical and non-canonical NLRP3 inflammasome activation.

Another relevant issue concerns the inhibition of NLRP3 downstream signaling through the blockade of IL-β receptor. In this respect, several lines of evidence have shown that treatment with IL-β receptor antagonists (i.e., anakinra) exerted beneficial effects in patients with immune-mediated inflammatory diseases (i.e., rheumatoid arthritis, ankylosing spondylitis, and gout) (73). In addition, anakinra reduced postoperative inflammation and ameliorated postoperative ileus in mice (74), thus suggesting that IL-1β receptor blockade exerts beneficial effects during intestinal inflammation. Based on these findings, it is conceivable that both upstream and downstream inhibition of NLRP3 inflammasome could represent suitable pharmacological approaches for treatment of bowel inflammation. Therefore, the possible effects of the pharmacological blockade of IL-1β receptor in different animal models of colitis should be investigated, in order to find out the better strategy to inhibit the NLRP3 inflammasome pathway and counteract bowel inflammation.

In support of preclinical findings, clinical evidence has documented an increased IL-1β secretion from colonic tissues and macrophages of IBD patients, these patterns being correlated with the severity of disease, thus suggesting IL-1β as a key pro-inflammatory cytokine for the pathogenesis of IBDs (75). In addition, the activation of NLRP3 inflammasome in monocytes infiltrating the lamina propria and M1 pro-inflammatory macrophages isolated from intestinal specimens of IBD patients, seems to contribute to the disruption of epithelial barrier through a deregulation of tight junction proteins (i.e., claudin-1, claudin-2, and junctional adhesion molecule-A), as well as to induce epithelial cell apoptosis (76). In particular, NLRP3 inflammasome-induced IL-1β and IL-18 release from monocytes infiltrating the lamina propria alters tight junctions and promotes apoptosis in intestinal epithelial cells, and, subsequently M1 macrophages, recruited into the lamina propria, contribute to sustain the immune innate response, thus suggesting a detrimental role of NLRP3 inflammasome in IBD patients (76). Accordingly, a translation of preclinical evidence into clinical practice could allow a better understanding of protective/detrimental shift of NLRP3 in IBD patients.

In conclusion, given the heterogeneity of preclinical studies and the paucity of human studies, extensive investigations are awaited for better understanding the NLRP3 inflammasome functionality in non-immune and immune cells since early stages of intestinal inflammation, in order to clarify the relevance of NLRP3 inflammasome in the pathophysiology of IBDs. In addition, considering the close inflammasome-gut microbiota interplay, and that genetic NLRP3 mutations and environmental factors, altering the gut microbiota, are involved in the pathogenesis of IBDs, future investigations should be addressed to characterize the correlation between changes in enteric bacteria composition and hypo- or hyperfunctionality of NLRP3 inflammasome.

## Author Contributions

CP, GL-C, and LA wrote the first draft of the manuscript. CP prepared the figures. CB and MF revised the manuscript.

## Conflict of Interest Statement

The authors declare that the research was conducted in the absence of any commercial or financial relationships that could be construed as a potential conflict of interest.
